# Clinical Scores in Veterinary Medicine: What Are the Pitfalls of Score Construction, Reliability, and Validation? A General Methodological Approach Applied in Cattle

**DOI:** 10.3390/ani11113244

**Published:** 2021-11-13

**Authors:** Sébastien Buczinski, Antonio Boccardo, Davide Pravettoni

**Affiliations:** 1 Département des Sciences Cliniques, Faculté de Médecine Vétérinaire, Université de Montréal, Saint-Hyacinthe, QC J2S 2M2, Canada; 2 Centre d’Expertise et de Recherche Clinique en Santé et Bien-Etre Animal (CERCL), Faculté de Médecine Vétérinaire, Université de Montréal, Saint-Hyacinthe, QC J2S 2M2, Canada; 3 Dipartimento di Medicina Veterinaria, Università degli Studi di Milano, via dell’Università 6, 26900 Lodi, Italy; antonio.boccardo@unimi.it (A.B.); davide.pravettoni@unimi.it (D.P.)

**Keywords:** cattle, clinical scores, methodological approach

## Abstract

**Simple Summary:**

Clinical scores are practical tools that can be used in the daily management of cattle. Score building and validation are a challenge involving various methodological and statistical issues. This article provides a specific framework for clinical score building where the target condition can be assessed directly or indirectly. Practical examples are given throughout the manuscript in order to build new scores or to assess score robustness.

**Abstract:**

Clinical scores are commonly used for cattle. They generally contain a mix of categorical and numerical variables that need to be assessed by scorers, such as farmers, animal caretakers, scientists, and veterinarians. This article examines the key concepts that need to be accounted for when developing the test for optimal outcomes. First, the target condition or construct that the scale is supposed to measure should be defined, and if possible, an adequate proxy used for classification should be determined. Then, items (e.g., clinical signs) of interest that are either caused by the target condition (reflective items) or that caused the target condition (formative items) are listed, and reliable items (inter and intra-rater reliability) are kept for the next step. A model is then developed to determine the relative weight of the items associated with the target condition. A scale is then built after validating the model and determining the optimal threshold in terms of sensitivity (ability to detect the target condition) and specificity (ability to detect the absence of the target condition). Its robustness to various scenarios of the target condition prevalence and the impact of the relative cost of false negatives to false positives can also be assessed to tailor the scale used based on specific application conditions.

## 1. Introduction

Clinical scoring is used for various purposes, including specific conditions such as diarrhea, respiratory disease, lameness, and body condition score), and for various target applications e.g., pre-weaned calves, post-weaned calves, adult cows. A scoring system enables the scorers, including farmers, technicians, and veterinarians, to adopt a systematic approach.

With the progress of on-farm technology and precision medicine, the benefits of these human-based clinical scoring systems have been debated [1,2,3]. These clinical scoring systems are still used to validate several machine learning and precision medicine algorithms [3,4,5]. It is therefore important to understand their strengths and limitations when using them as reference standard tests or when relying on their use to determine specific morbidity events on farms. Although the process of clinical scoring system building can be similar to the building of scales used in various psychometric fields, there are some important differences in the way they are constructed and validated, as recently reviewed [6]. For this reason, the approach proposed in this paper is tailored to clinical scoring systems that can be used for food animals.

**Figure 1:**
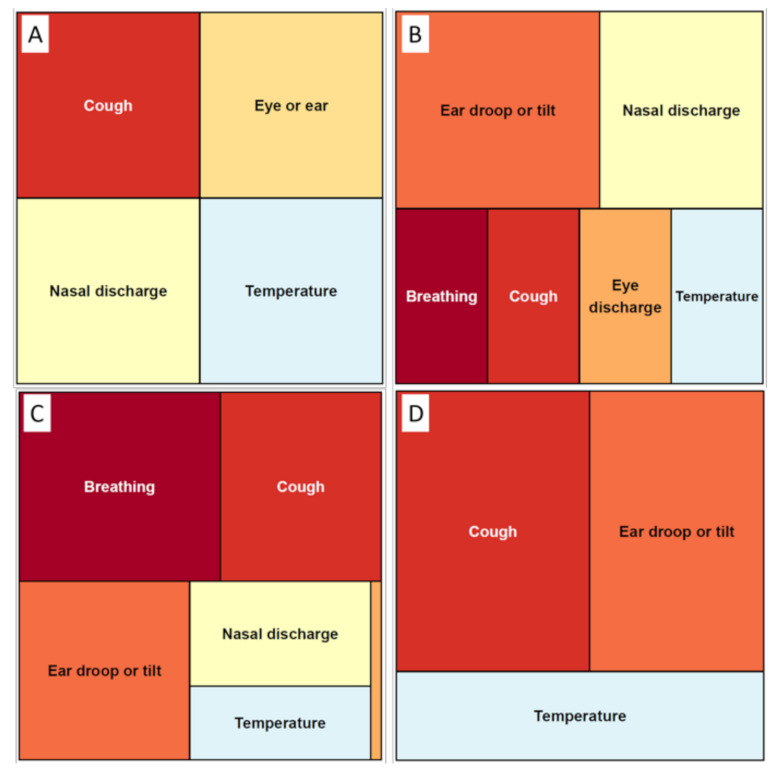
Treemap of the relative weights of different clinical signs included in various scoring systems used to detect bovine respiratory disease complex in calves. The size of the square is proportional to the specific clinical sign weight in the Wisconsin calf respiratory score (**A**) [7], California score (**B**) [8], Québec modified California score (**C**) [9], and Québec veal calf respiratory score (**D**) [10]. The map clearly shows the variable signs included and the relative impact on the total score of the same clinical sign.

There are several scoring systems for relatively similar purposes. For example, at least three different scoring systems are routinely used to evaluate respiratory diseases in pre-weaned dairy calves [7,8,9] or in veal calves [10], as indicated in Figure 1.

**Figure 2:**
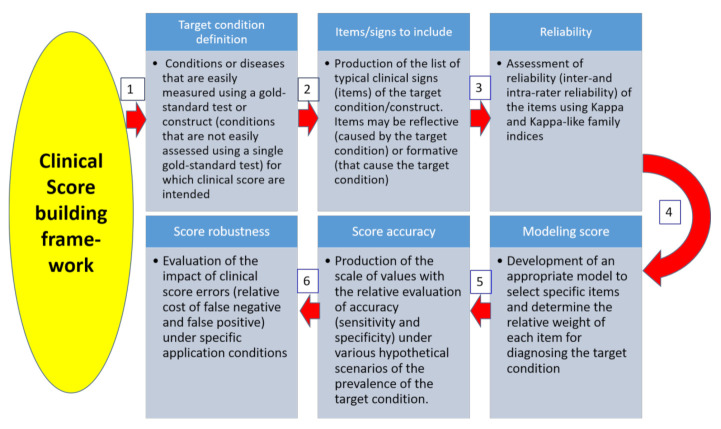
General framework to establish and validate a clinical scoring system in veterinary medicine. Our 6-step framework is depicted with an initial definition of the target condition (**1**), the potential items of interest (**2**), and their reliability (**3**). The modeling approach then determines the adequate repartition between and within item weights (**4**). The accuracy of the score is determined in step (**5**). The optimal threshold selection is then adapted to the specific conditions where the test is intended to be used (**6**). Score users also need to be adequately trained.

Other scoring systems exist, for example, to determine calf diarrhea [7], lameness [11], body condition [12], and pain [13]. In these cases, it is important to know the pros and cons of the various scoring systems in order to use the best one for a specific setting. On the other hand, when there is no specific scoring method, it is important to know how to construct a scoring application. This manuscript highlights the possible objectives of a scoring system and the methodological procedure that should be constructed and validated, including the adequation between the score and what it is supposed to measure together with the reliability issues (Figure 2).

Section 2 defines the notion of construct, which is the condition or status that the score is supposed to measure. In Section 3, we highlight the problems of measuring the different items that make up the score and look at how to determine the optimal combination used in the score. Section 4 outlines the determination of score accuracy and level of discrimination. Finally, Section 5 focuses on the most difficult constructs to define which involve similar challenges as those found in psychometric and behavioral fields.

## 2. Condition That Needs to Be Measured: Notion of Construct

Scoring systems are generally designed with an initial target condition to diagnose that can be measured objectively using a gold-standard test. This gold standard test cannot generally be used routinely due to cost, invasiveness, convenience, or the need for a specialized lab. This is why a more practical first decision-making step is needed. For example, the lameness score based on visual gait analysis could be initially compared with the weight repartition forces on the ground and specific gait analysis measurement [14]. Again, the neonatal calf diarrhea scoring scale can be objectively determined in calves using metabolic cages that assess fecal output and dry fecal matter [15]. Other target conditions that need to be measured may be more difficult to describe accurately. This is also common in human medicine, e.g., psychology. In this situation, the different scores are used to assess a “construct” [16].

The term construct is generally used for non-easily observable characteristics in order to identify the subject of measurement. Various types of constructs where no specific affordable gold standard reference exists are also encountered in cattle. The bovine respiratory disease complex (BRD), which is a complex mixture of infectious agents affecting the upper and lower respiratory tract, is a common example in cattle medicine [17]. Stress conditions and the welfare status of animals are also construct categories that are complex to determine and define precisely [3].

The first challenge is thus when the construct cannot be easily measured or defined. In this case, the construct is a “latent variable” that cannot be measured accurately. Each scale or score aims to regroup different items that can be determined objectively or subjectively and are associated with the construct. The construct validity is challenging to define. As a rule, construct validity determines the degree to which a specific test successfully measures what it claims to test. It can, for example, be based on a specific panel determination that assesses how adequately the items included in the scale are associated with the construct to be measured. In terms of diagnosing bronchopneumonia in calves, an expert panel can determine several clinical signs that may be of interest for the clinical diagnosis.

It is then crucial to determine other components for the validity of the score before its practical implementation. Validity is difficult to assess since it encompasses various dimensions. The content validity of the score is different from the construct validity. The content validity assesses whether the test is fully representative of what it is supposed to measure. The difference between construct and content validity is not easy to distinguish. However, the construct validity is associated with the definition of the construct that the test is supposed to measure. On the other hand, the content validity examines how the test dimensions fully represent the construct characteristics [16].

When the score aims to evaluate a more complex construct that is difficult to determine with a reference standard test (e.g., pain or stress), it is impossible to determine its accuracy using the method previously presented. A practical example of this is a score that assesses the stress or welfare of calves. In these situations, the construct has multidimensional aspects in the sense that various environmental conditions and animal-based aspects (clinical signs, behaviors, or biomarkers) may be indicative of stress without a specific combination of tests considered as a gold standard test.

Another dimension not accounted for in the literature is the quantitative assessment of the level of stress or welfare. In this case, the condition of interest should be gradated, which is another specific aspect in this condition (vs. tests used for detecting a disease or a condition (present vs. absent)). The steps for building this type of scale are similar (with some adaptations) to those in the social and behavioral fields [6]. The three steps for building the scale are item development and then scale development and validation. Item development aims to identify the domains that need to be assessed for the construct assessment. Then, specific items are built that can be based on biological and behavioral assessments. Once these items have been developed, content validity is helpful to determine the adequation between the item and the domain of interest. The content validity examines the content representativeness and relevance, as well as the technical quality of measurement. These aspects should be assessed by experts (ideally different ones from the initial team building the scale) using various quantitative and qualitative tools. The scale is then developed after pretesting questions and administering the scale in a representative sample of target animals.

## 3. How to Measure Items Present in the Score?

First, as with any measurement, the following should be defined: (i) the object of measurement (in this specific case the calf or the cow), (ii) the property/item that is measured (weight, rectal temperature, body condition score, specific behavior), and (iii) the values that could be used to determine this property/item (e.g., kg, °C, different strata of body condition score (BCS)). Objective items can be measured without a clinician’s or operator’s interpretation. For example, calf weight determined with a scale is an objective measurement that only depends on the scale’s reliability. Nevertheless, the same variable (weight) can be associated with other sources of measurement error if, for example, weight is estimated using heart girth measurement [18]. In this case, in addition to tape measurement errors, other sources of uncertainty could be related to the relationship between the heart girth and body weight and how the operator applies the tape. Some items are obviously subjective (i.e., they depend on the rater), such as evaluating the body condition score in cows even using guidelines. However, many objective items are, in fact, partly subjective. For example, the rectal temperature measurement may not only be based on the type of thermometer used, but also on how the probe is inserted [19], which raises the same type of concern as tape application for heart girth measurement. Imaging methods are also affected by operator technique and image interpretation, such as quantifying lung lesions using thoracic ultrasonography in cattle [20]. This is another example of apparently objective measurements (i.e., quantifying lung lesions with a specific measure) that are in reality subjective measurements when the operator is potentially an important source of error. However, this does not mean that subjective measurements should be excluded from scale construction, but that their intra- and inter-rater reliability should be accounted for when developing the score and scale.

**Figure 3:**
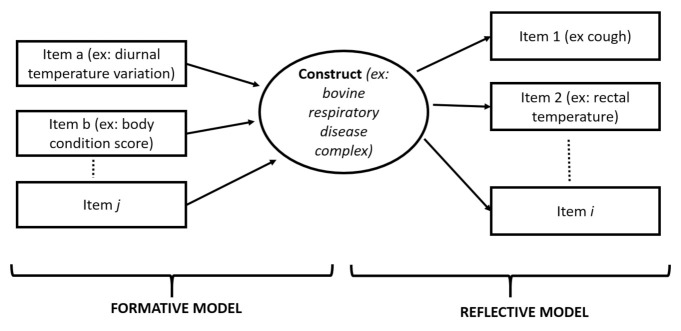
General framework to determine the specific items to be included for a specific construct or disease. Different items can be selected to assess a specific construct or determine a target condition. Reflective items are generally a reflection of the target condition or part of the description of the condition. In this example, cough or rectal temperature could be considered as part of the description of signs of respiratory disease (or the construct can cause them). By contrast, formative items can be considered as more causal items. They can cause the construct. In this example, diurnal variation of external temperature could be associated with an increased risk of bovine respiratory disease complex (BRD) but is not actually caused by BRD. For some items, it may be more difficult to determine both their formative and reflective roles. For example, the BCS could either be formative (e.g., calves with a low BCS could be at higher risk of being sick) or reflective (long disease duration may lead to a low BCS).

The conceptual framework that illustrates how the items are related to the construct to measure is critically important. The two different frameworks used are either reflective or formative (Figure 3). In a reflective model, the items are a specific manifestation of the construct to be measured. With a formative model, the construct is the result of the item to be evaluated. A practical way of determining whether an item is formative or reflective is to determine whether a change in the construct would be associated with a change in the item manifestation. Complex constructs can be based on a mixed formative and reflective model. As a practical example in a scale assessing BRD in weaned calves, Maier et al. [21] included reflective items (sunken eyes, cough, breathing pattern, and rectal temperature) and formative items (e.g., body condition scoring, diurnal temperature fluctuation). A change in BRD status or severity might easily impact on reflective items, whereas formative items would not (Figure 3). This difference in items is not clear-cut since some formative items may in fact be reflective (in the previous case, the body condition score can be viewed as either a formative item (with low BCS calves at higher risk of being affected) or reflective (duration and severity of the respiratory condition lead to a lower BCS).

### 3.1. Items Selected and Classification of the Different Possible Categories of Items

The association between the different clinical signs or factors included in the score needs to be detailed. For example, adding highly correlated clinical signs would not be useful from a monitoring basis. If one clinical sign is highly correlated with another, knowing one of the clinical signs would implicitly mean that the second sign is also known (because of the high correlation between the signs). The information gained from knowing one rather than two signs would therefore not improve the monitoring capacity in a large population. However, using correlated questions could be essential for complex constructs with various dimensions in order to gather information that differentiates between some patients and highlights a single or multidimensional domain of the score [6].

A precise selection of the most practical signs is essential as targeted score-users measure their reliability. The stratification of the items to be scored is another major topic of interest. The stratification process should be clearly defined with clear mutually exclusive definitions. Several items scored in various cattle scoring systems do not meet these criteria. In the Wisconsin score system, nasal discharge is noted as normal serous discharge (score = 0), small amount of unilateral cloudy discharge (score = 1), bilateral, cloudy, or excessive mucus discharge (score = 2), and a copious bilateral mucopurulent discharge (score = 3). What would happen if a calf only showed a small amount of mucous discharge? And what is a “small amount” of unilateral cloudy discharge, or how should “copious” be objectively defined? Some raters will not have the same conception of “small amount” or “copious.” Pictures associated with the scorecards that explain the score are helpful but not self-explanatory enough to ensure that the definition is reliable within and between raters. This may explain a significant part of the score variability that is not attributable to the measurement object (calf) but to the limited reliability between scorers.

This is only one example of the reliability issues when implementing scoring systems in practice, especially when the score is a specific outcome for monitoring different interventions or management changes. Improvement in definitions using more objective measurements (e.g., the quantification of the importance of the discharge) or the duration of the observation period to define cough frequency could be used to decrease rater subjectivity. Specific training can also improve consistency among scorers.

Using multiple categories for one item assessment is also useful for the gradation of clinical sign modifications. The natural drawback of using multiple categories is that it is associated with an inflated risk of altered reliability due to more choices in the item scale [11]. This should also be accounted for when proposing the different categories of items. Another challenge is to determine how the clinical sign is associated with the construct, its relative importance in relation to other signs included in the score, and the relative weight of the different categories of the scale within the same clinical sign.

#### 3.1.1. Intra and Inter-Rater Reliability

Reliability is a key issue for any clinical measurement. When the various items to be rated have been selected, their test-retest reliability (first by the same rater and then by different raters) should be assessed. Depending on the type of item to be rated, several complementary approaches can be used [22]. Kappa and kappa-like family indices are generally used to differentiate agreements that occur purely by random chance versus true agreements. The difference between the multiple types of agreement indices is generally based on different definitions in agreement by chance as well as the way a partial agreement (in ordinal scale) is accounted for [23]. We only focus on the most common parameters used.

#### 3.1.2. Two Raters Categorical Scale

Cohen’s kappa [24] is by far the most commonly reported agreement parameter in veterinary medicine and many other medical fields. The general framework for kappa calculation is to correct the percentage of agreement between the two raters corrected with the percentage of the agreement only due to chance (i.e., the percentage of agreement that would be obtained if the raters’ ratings were independent of each other). This concept is explained in Table 1. When *n* total subjects are scored by two raters (1 and 2), the results of the tests can either be (1,0), the subjects are cross-classified based on the test results probability with r*_ab_* (a = test result by rater 1 (0 or 1), b = test result by rater 2 (0 or 1)) and r_11_+ r_10_ + r_01_+ r_00_ =1.

The observed raw probability of agreement (Pa) between the two raters is

(1) $$Pa=\left(r_{11}+r_{00}\right)$$

The chance agreement (Pc) is defined as the probability of observing agreement if the raters were independent:

(2) $$Pc=\left(r_{11}+r_{10}\right)\times \left(r_{11}+r_{01}\right)+\left(r_{01}+r_{00}\right)\times \left(r_{10}+r_{00}\right)$$

The kappa is then simply calculated using the following formula correcting *Pa* with *Pc*.

(3) $$K=\frac{Pa-Pc}{1-Pc}$$

The standard deviations for calculating 95% CI using the frequentist approach is [24]:

(4) $$SD\left(K\right)=\sqrt{\frac{Pa\left(1-Pa\right)}{{\left(1-Pc\right)}^{2}}}$$

The 95%CI of the K value can therefore be obtained using the standard normal distribution of the K with K +/− 1.96 SE when α error is 0.05. This can further be extended for other values of α with the 100* (1 − α) confidence intervals for K computed as:

(5) $$K+/-z_{\alpha /2}\times \frac{SD\left(K\right)}{\sqrt{n}}$$

The interpretation of the confidence intervals is not clinically intuitive and is generally falsely interpreted as Bayesian credible intervals. Determining K confidence intervals has been criticized from a frequentist perspective [25]. Benchmarking these intervals is not evident under the frequentist framework despite some recently proposed approaches [23,26].

These concepts can be easily illustrated when two raters scored a population of 100 calves using a specific scoring system (see Supplementary File). It is straightforward to extend the kappa calculation for multiple categories (>2). In this situation, a weighted kappa (K*w*) can also be calculated if the categories are ordinal, indicating that a different weight is applied depending on putting more weight on the error in two distant categories versus differences in adjacent categories [23].

The Bayesian version of the K with readily applicable credible intervals calculated without specific parametric assumption can also be obtained using the same equation for calculating K but considering that each cell content of the 2 × 2 table has a gamma (Γ) distribution Γ (k, θ) with shape k and relatively large scale θ. The shape is directly obtained from the numbers in the initial classification (*n11*, *n10*, *n01, n00*), adding 1 (*n11* + 1, *n10* + 1, *n01* + 1, *n00* + 1) since these variables are assumed to follow a Dirichlet distribution. The scale θ is generally given a >1 value (e.g., 2), which helps to extend the ranges of possible distribution. The K values are then obtained from multiple iterations of the process to obtain the density of K distribution [27].

The Bayesian version of agreement and reliability measures is currently quite complex as there is no easy-to-use calculator (Supplementary File). The reliability measures generally rely on more extensive coding ability and, although promising, are only used by few researchers [27,28].

#### 3.1.3. Multiple Raters

The agreement process can be extended with multiple-rater scoring by generalizing the previous concept of agreement by chance based on Fleiss’s extension of Scott’s π statistics (known as Fleiss’ kappa K*_F_*) [23]. Scott’s π statistic is slightly different from K since it accounts for the mean predicted probability between *r* different raters (r ≥ 2) scoring an object in *q* different categories.

(6) $$K_{F}=\frac{Pa-Pc|\pi }{1-Pc|\pi }$$

With:

(7) $$Pa=\frac{1}{n}\;\overset{n}{\underset{i=1}{\sum }}\overset{q}{\underset{k=1}{\sum }}\frac{r_{ik}\;\left(r_{ik}-1\right)}{r\left(r-1\right)}$$

where r_ik_ is the proportion of the r raters affecting to the *i*th subjects the k categories and with:

(8) $$Pc|\pi =\overset{q}{\underset{k=1}{\sum }}{\hat{\pi }}_{k}^{2}$$

and

(9) $$\hat{\pi_{k}}=\frac{1}{n}\overset{n}{\underset{i=1}{\sum }}\frac{r_{ik}}{r}$$

The confidence intervals can be obtained from a specific determination K*_F_* standard error (for details see [23]). Although kappa-like indices are still widely used, they have various drawbacks known as kappa paradoxes, especially when the calculated Pc is very high and where K is close to 0 despite a high crude percentage of agreement [29]. New indices, such as Gwet’s agreement coefficients (AC), have been explored to avoid the pitfalls involved in calculating Pc [23]. The AC parameters complement traditional K statistics [26,30].

There are many other agreement/reliability indices, but they are beyond the scope of this article. However, a common situation when testing the reliability of categorical item measurement is missing data, e.g., the different scoring sessions where different raters assess different animals [31]. In this specific case, not all the rater–animal pairs are available. Since they only use the complete dataset, calculations of kappa and kappa-like statistics (e.g., Fleiss kappa) are not robust to missing data. In this situation, Krippendorff’s alpha is the most robust approach given that it does not only exploit the complete pair-rater scores [32]. See [23,32] for information on how to calculate this reliability index and associated confidence intervals. The Gwet AC parameters are also robust to missing data.

In summary, the agreement assessment between raters assessing a categorical scale is complex and can generally not be performed simply by reporting one parameter. Reporting multiple aspects of the agreement with raw agreement and chance-corrected agreement parameters is recommended. However, the specific association of which chance-adjusted agreement parameter to report is still an open issue.

#### 3.1.4. Numeric Items

The previous calculations formerly adapted to categorical scales do not work for quantitative items that can be measured to assess the health of calves or cows. In these situations, the measured item (M) is generally a numerical value that quantifies the true value of interest (η) with a specific error (ε). This relationship can therefore be written as follows:

(10) M=η+ε

The specific ε term can further be classified as the error or measurement device, error due to operator, or any other cause of error. Reliability concepts applied to these measurements are associated with the variance (*σ*^2^) of *i*th repeated measurements M by different raters, time or specific conditions (M*i*), and the specific error term (ε*_i_*). For example, the true internal temperature (η) can be assessed by the rectal temperature measurement (M*i*) by *i* different persons or using *i* different thermometers. The aim of the reliability parameters is generally to quantify the variance of the true measure ($\sigma^{2}\left(\eta \right)$) versus the general variance of the error ($\sigma^{2}\left(\varepsilon_{i}\right)$), which can further be simplified as:

(11) $$Reliability=\frac{\sigma^{2}\left(\eta \right)}{\sigma^{2}\left(\eta \right)+\sigma^{2}\left(\varepsilon_{i}\right)}=\frac{\sigma^{2}{}_{patient}}{\sigma^{2}{}_{patient}+\sigma^{2}{}_{error}}$$

Therefore, the reliability assesses the relative error versus the true value variance, or the ratio of the variance of interest divided by the variance of interest plus the unwanted (noisy) variance. This family of reliability parameters is also called intra-class correlation (ICC) measures [33]. There are multiple types of ICC that depend on how error variance is partitioned. These include combinations between one-way or two-way random, two-way mixed effects, and absolute (ICC(A)) vs. consistency (ICC(C)) agreement (for details see [33]).

#### 3.1.5. Benchmarking Reliability Parameters

Once a reliability value has been obtained for either categorical or numerical items, it is crucial to determine whether these values are compatible with the intended use of the score or item assessment. Most benchmarks have not been extensively validated but are opinion-based [34]. For example, is not easy to know whether in practice a kappa of 0.8 is different from a kappa of 0.6. Based on the calculation difference, using a single agreement reliability parameter is not enough to assess all the dimensions of agreement. Several differences may be observed and should be discussed regarding the intended use of the score or item [23]. Although general benchmarks have been reported for numerical items using ICC measures [33], they have not been thoroughly validated and should be used in the light of the specific study context.

## 4. How Can the Best Combination of Items Be Determined for Use in a Score?

### 4.1. Score Building to Determine the Relative Weight between and within Items That Are Assessed to Measure a Simple Construct

Once different clinical items included in the score have been validated with their inter-and intra-rater variability, the next step is to determine the strength of association between all items and the different categories with the construct to be measured by the different items and their values. Linear, generalized linear model, or survival analyses possibly accounting for correlations between the patients (e.g., herd aggregation level) should thus be built. A specific framework for diagnostic or prediction models has been proposed with the transparent reporting of a multivariable prediction model for individual diagnosis or prognosis (TRIPOD) statements [35]. The general framework of reporting can be explained for a specific outcome Y, with X_1_,…,X_n_ clinical signs or specific covariates with respectively (k, …, n_k_ categories) and *u* the random error (if any) in the form:

(12) $$g\left(Y\right)=\beta_{0\;}+\beta_{11}X_{11}+\ldots +\beta_{1k}X_{1\left(k-1\right)}+\ldots +\beta_{n1_{n}}X_{n1}+\ldots +\beta_{nk_{n}}X_{n\left(k_{n}-1\right)}+u$$

The link function between the construct or condition to diagnose (Y) and the different covariates depends on the type of construct and the way it is assessed. When the construct is a binary condition that can be diagnosed with an accurate reference standard test (considered as a gold standard), the binomial link with logistic regression to determine the probability of the presence of the disease (*p*, which is the probability that the gold standard test is positive) is the natural choice, and this was exploited in a study using a specific BRD case definition [8]. Survival analysis models are generally recommended when the time to event outcome is of interest.

In many clinical conditions, there is no affordable 100% accurate diagnostic test to determine the animal status (Y). For example, in the diagnosis of BRD where, except for necropsy, no reference standard test is accurate enough to be exempt from classification error. In these conditions, there are three alternatives: (i) choosing a proxy of the target condition which is at risk of bias due to imperfect accuracy (e.g., for BRD: thoracic ultrasonography); (ii) using a composite reference standard test can be a solution (e.g., for BRD, using an association between thoracic ultrasound, serum haptoglobin, and bronchoalveolar lavage) although this has its own risk of bias [36]; and finally (iii) accounting for the imperfect accuracy of a test that can be done in a practical setting to define the latent status of the animal [37]. The modeling approach is similar in the first two conditions since the reference test is defined as positive (Y = 1) and negative (Y = 0) animals.

However, the framework is different when the true status (target condition or construct) is considered unknown (latent) but can be described based on the known accuracy of the imperfect test used. In this case, the probability that an animal has an outcome (*p* = *p*(Y = 1)) can be associated with the probability that the animal reference standard test is positive (pT+) or negative, accounting for test sensitivity (Se) and specificity (Sp) as follows:


(13) $$g\left(Y\right)=logit\;p=\beta_{0\;}+\beta_{11}X_{11}+\ldots +\beta_{nk_{n}}X_{nk_{n}}$$

(14) $$p_{T+}=p*Se+\left(1-p\right)*\left(1-Sp\right)$$

Therefore, the imperfect accuracy of the test means that unbiased model characteristics can be determined when the outcome is measured with error [8,38].

#### Internal vs. External Validation of the Model

The model needs to be validated especially in terms of refining the estimates of β parameters in order to account for potential shrinkage when applied to a new population. This topic is also a fundamental research area in biostatistics that encompasses the current manuscript [39,40]. Initial model selection is always associated with overestimating model accuracy and discrimination. This is because the model performance is higher in the dataset where the original model was developed than in a new dataset (“testimation” problem).

The value of the different β should then be the basis for calculating the individual weights of model predictors. It is essential not to use the transformation of the estimates as a score weight (e.g., OR) because linearity between the predictors and the construct is only valid on the assumption of a g(.) link and not with the odds ratio derived from the coefficients in a logistic regression approach [41]. For practical purposes, the β are generally multiplied by a common number (e.g., 5 or 10) and rounded to obtain the full score and items.

### 4.2. Measurement of Complex Construct

Measuring a complex construct with a multidimensional aspect is totally different from the precedent approach. For example, building a tool to assess the welfare of calves is not comparable with a standard reference test, which would only capture one specific dimension of the construct. Measuring serum cortisol, for example, as an indicator of stress (e.g., proxy of the target condition to measure) would be a welfare dimension. However, the definition of welfare includes measuring a specific biomarker. In this situation, building the score would have a different assessment framework and other specific statistical parameters that are outside the scope of this article [6].

## 5. Accuracy of the Score

### 5.1. Case 1: The True Status of the Construct Can Be Determined (Directly or Indirectly)

Once the ideal repartition between the various items and the various categories within the same items have been determined, it is then necessary to determine how to implement the score in practice with a specific recommendation concerning the thresholds. For the diagnosis of a dichotomous condition, the accuracy of the scoring system could be determined using the ability to diagnose animals with the target condition (sensitivity) and the ability to diagnose non-affected animals (specificity).

**Figure 4:**
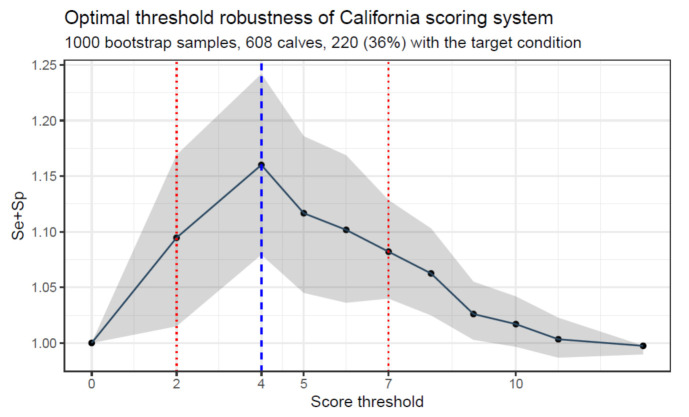
Determination of the optimal threshold based on a specific score. This figure represents the California respiratory score threshold for detecting ultrasonographic lung consolidation (target condition defined as a maximal consolidation depth ≥ 1 cm) in 608 pre-weaned calves from 39 different dairy herds [9]. The accuracy of the score is presented in the y-axis with the sum of sensitivity (Se) and specificity (Sp) of the score depending on the threshold (≥ threshold to define a positive score, < for a negative score). The dots represent the observed value of the score, and the 95% confidence interval band was obtained after 1000 bootstrap estimates. Even though the threshold of ≥ 4 is numerically associated with the highest accuracy (dashed blue line), there is no evidence that this threshold is better than any threshold between 2 and 7 (dotted red lines) due to the confidence interval width.

The optimal combination of sensitivity and specificity can be determined using different decision thresholds. Minimizing the total misclassification rate, and thus maximizing the Se+Sp sum (Figure 4), is generally the default in many situations for diagnostic test accuracy assessments [42]. However, this selection is arbitrary and does not account for the test’s intended use and the differential value of false-positive versus false-negative cases. In some situations, missing truly affected animals is more deleterious than the false-positive classification of non-affected animals.

**Figure 5:**
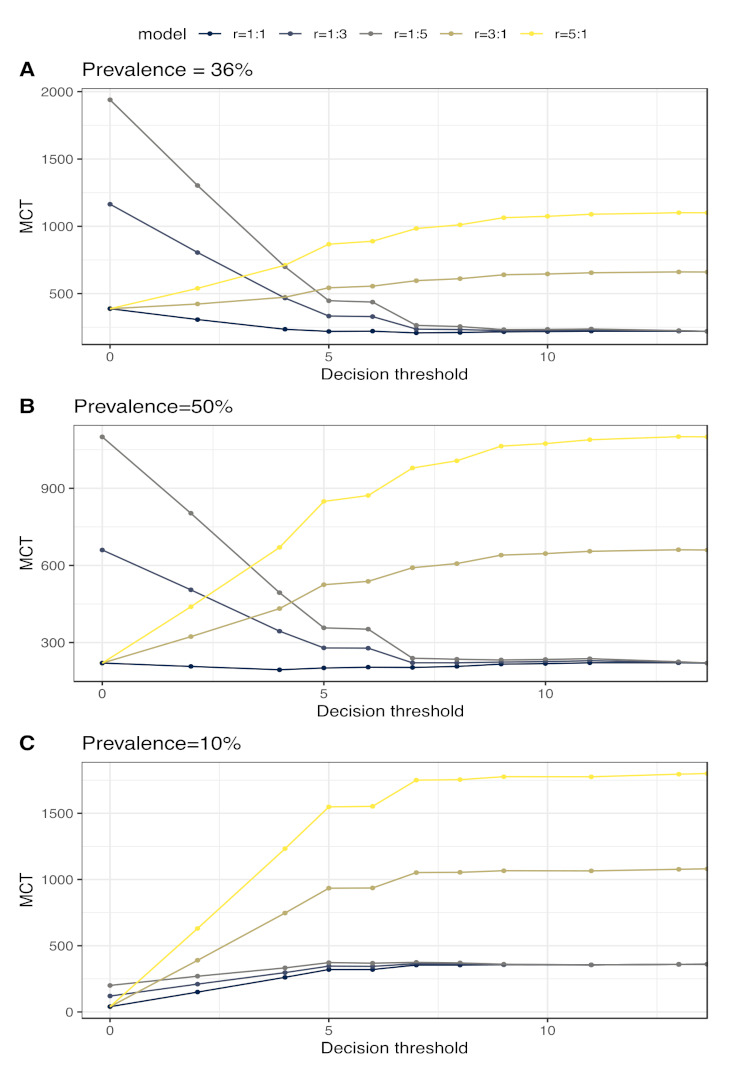
Misclassification cost-term (MCT) analysis of a specific score to determine lung consolidation. The same dataset as in Figure 3 was used to determine the misclassification cost term using: (**A**) the original prevalence of the target condition in the dataset (36% of calves were detected with ultrasonographic lung consolidation depth ≥ 1 cm); (**B**) a prevalence of 50%; and (**C**) a prevalence of 10% based on internal resampling. Different false negative (FN): false positive (FP) cost ratios (*r)* are indicated. In the first scenario, *r* was considered as 1:1, meaning that FN and FP had the same impact. Two scenarios where FN calves were considered more costly than FP calves with 3:1 and 5:1 *r* values. Finally, two scenarios considered that false-positive calves were more costly than FN cases with 1:3 and 1:5 *r* values. The 5 MCT curves are then derived to determine the score threshold robustness for various settings. The optimal threshold minimizes the y-axis value.

A specific example of this is bronchopneumonia in calves, where not finding affected animals would have a more deleterious animal health impact in terms of the risk of incomplete cure and negative outcome as against recommending treatment in a non-affected calf. This relative cost would depend on the type of cost assessed. Using the same BRD example but from a public health/rational use of antimicrobials, the associated risk of antimicrobial use in non-affected animals would have a significant impact. The exact definition of “cost” may depend on the context (farm, country, etc.). A complete assessment of costs can therefore not be easily performed. It is thus important to test various plausible scenarios that cover broad but plausible situations (Figure 5).

Various test applications in terms of the possible prevalence of the target condition (*p*) and the relative cost of false-negative versus false-positive (FN:FP) cases (*r*) can be determined by calculating the misclassification cost term (MCT), which should be minimized [43]. The MCT calculation can be written as:


(15) $$MCT=\left(1-p\right)*\left(1-Sp_{t}\right)+r*p*\left(1-Se_{t}\right)$$

for the various possible *t* thresholds of decision. The MCT is not in itself an extensive cost analysis examination but rather a practical way to address the robustness of the cut-off depending on different plausible (“what if”) scenarios of the target condition prevalence and *r* values. If the minimum MCT area is very variable depending on *p* and *r*, the recommendation for applying a common threshold should be determined more in depth before applying the test in practice (Figure 5). When the MCT reaches its minimum independently of *p* and *r*, that threshold is considered a relatively robust threshold which can be applied in the various conditions found in practice. However, robustness is not equivalent to accuracy since a robust threshold can have a limited accuracy.

### 5.2. Case 2: More Complex Construct

Because it is beyond the scope of this paper, we have not discussed the best way to deal with the items depending on the nature of their quantification, the dimension they are supposed to measure, and the impact of the nature of the item (i.e., formative vs. reflective) on scale building (for details see [6]).

In conclusion, clinical scores have an important role in determining various health and welfare conditions for food animals. The construction, reliability and, validation of the score depend on the construct or target condition of interest. We have focused on constructing or targeting conditions that can be determined directly (using a gold standard test) or indirectly (using an imperfect reference standard test). The reliability of the possible items to be included, the acquirement of the relative item weight in the final score, and the determination of the decision threshold to apply based on various contexts have been explained. Future work should focus on the internal and external validation of the decision threshold in a different population from where it was initially derived to improve its robustness. Although this paper has focused on bovine health and welfare condition monitoring, the proposed framework could be extended to any species of veterinary interest since the same concepts and rules would be applicable. It is also important to mention that automatic data collection is also a promising area of research in the detection of animal health problems with the implementation of a machine learning strategy and artificial intelligence technique. We chose to focus on clinical signs that can be detected by farmers or veterinarians, but automatic behavior or anomaly detection is definitely an important potential evolution of disease detection.

## Figures and Tables

**Table 1:** Example of 2 × 2 table results based on a dichotomous scale obtained by two raters scoring *n* different animals for an item.

	Test = 1 for Rater or Test 2	Test = 0 for Rater or Test 2	
**Test = 1 for rater or test 1**	*n*_11_ = *n*×*r*_11_	*n*_10_ = *n*×*r*_10_	
**Test = 0 for rater or test 1**	*n*_01_ = *n*×*r*_01_	*n*_00_ = *n*×*r*_00_	*n*

## Data Availability

Not applicable.

## Supplementary Materials

The following are available online at https://www.mdpi.com/article/10.3390/ani11113244/s1, Supplementary File: Example of agreement calculation with frequentist method and Bayesian method.
