# Supplementary material for: Clinical Scores in Veterinary Medicine: What Are the Pitfalls of Score Construction, Reliability, and Validation? A General Methodological Approach Applied in Cattle
Source: Animals (Basel). 2021 Nov 13;11(11):3244. doi: 10.3390/ani11113244 (PMC8614512; doi:10.3390/ani11113244)

Supplementary file

Two different raters scoring 100 calves with a specific score chart. Twenty calves were found positive for both raters and 70 calves were found negative for both raters. 10 calves obtained discrepant results between raters.

	Rater 2, test +	Rater 2, test -	
Rater 1, test +	20	3	
Rater 1, test +	7	70	100

Frequentist approach:

$$Pa = (20 + 70)/100 = 0.9$$

$$Pc = \left[\frac{(20 + 3) * (20 + 7)}{100 * 100} + \frac{(70 + 3) * (70 + 7)}{100 * 100}\right] = 0.624$$

Then K is simply obtained by:

$$K = \frac{0.9 - 0.624}{1 - 0.624} = \frac{0.276}{0.376} = 0.73$$

The SE(K) is 0.08 with K95% CI: 0.58-0.89.

Bayesian approach

The Bayesian approach is summarized with specific coding approach as a supplemental file for this specific example. It gives slightly different estimations with K=0.70 and 95%BCI (0.53-0.84). The probability that the true K >0.7 is 51.5% (20,000 iterations with 5,000 burn-in, 3 different chains). Both estimates and their spread under frequentist (red) and Bayesian (blue) framework are presented below.

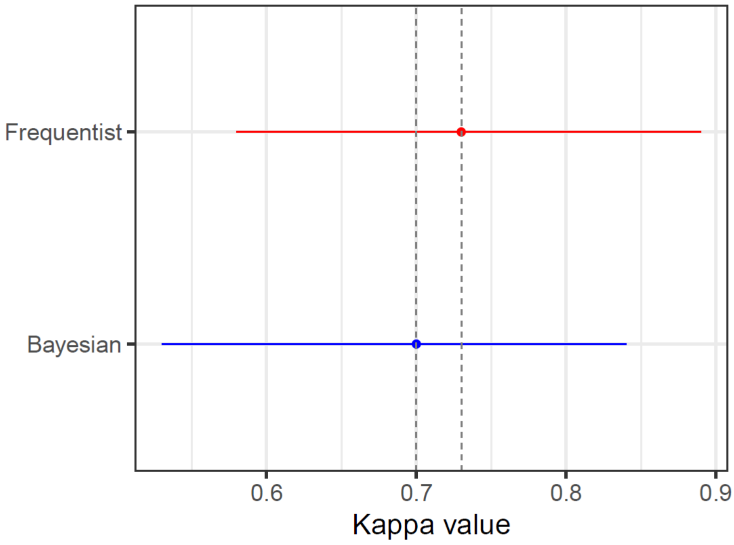

Supplement: Supplementary file 1 [file animals-11-03244-s001.zip › animals-1418403-supplementary.pdf]
